# Comprehensive dosimetric and clinical evaluation of lexicographic optimization-based planning for cervical cancer

**DOI:** 10.3389/fonc.2022.1041839

**Published:** 2022-11-16

**Authors:** Sara Trivellato, Paolo Caricato, Roberto Pellegrini, Gianluca Montanari, Martina Camilla Daniotti, Bianca Bordigoni, Valeria Faccenda, Denis Panizza, Sofia Meregalli, Elisa Bonetto, Stefano Arcangeli, Elena De Ponti

**Affiliations:** ^1^ Medical Physics Department, Azienda Socio Sanitaria Territoriale (ASST) Monza, Monza, Italy; ^2^ Department of Physics, University of Milan, Milan, Italy; ^3^ Global Clinical Science, Elekta AB, Stockholm, Sweden; ^4^ Department of Physics, University of Milan Bicocca, Milan, Italy; ^5^ School of Medicine and Surgery, University of Milan Bicocca, Milan, Italy; ^6^ Department of Radiation Oncology, Azienda Socio Sanitaria Territoriale (ASST) Monza, Monza, Italy

**Keywords:** lexicographic optimization, automated planning, cervical cancer, VMAT (volumetric modulated arc therapy), plan quality, plan comparison

## Abstract

**Aim:**

In this study, a not yet commercially available fully-automated lexicographic optimization (LO) planning algorithm, called mCycle (Elekta AB, Stockholm, Sweden), was validated for cervical cancer.

**Material and methods:**

Twenty-four mono-institutional consecutive treatment plans (50 Gy/25 fx) delivered between November 2019 and April 2022 were retrospectively selected. The automatic re-planning was performed by mCycle, implemented in the Monaco TPS research version (v5.59.13), in which the LO and Multicriterial Optimization (MCO) are coupled with Monte Carlo calculation. mCycle optimization follows an *a priori* assigned priority list, the so-called Wish List (WL), representing a dialogue between the radiation oncologist and the planner, setting hard constraints and following objectives. The WL was tuned on a patient subset according to the institution’s clinical protocol to obtain an optimal plan in a single optimization. This robust WL was then used to automatically re-plan the remaining patients. Manual plans (MP) and mCycle plans (mCP) were compared in terms of dose distributions, complexity (modulation complexity score, MCS), and delivery accuracy (perpendicular diode matrices, gamma analysis-passing ratio, PR). Their clinical acceptability was assessed through the blind choice of two radiation oncologists. Finally, a global quality score index (SI) was defined to gather into a single number the plan evaluation process.

**Results:**

The WL tuning requested four patients. The 20 automated re-planning tasks took three working days. The median optimization and calculation time can be estimated at 4 h and just over 1 h per MP and mCP, respectively. The dose comparison showed a comparable organ-at-risk spare. The planning target volume coverage increased (V_95%_: MP 98.0% [95.6–99.3]; mCP 99.2%[89.7–99.9], p >0.05). A significant increase has been registered in MCS (MP 0.29 [0.24–0.34]; mCP 0.26 [0.23–0.30], p <0.05) without affecting delivery accuracy (PR (3%/3mm): MP 97.0% [92.7–99.2]; mCP 97.1% [95.0–98.6], p >0.05). In the blind choice, all mCP results were clinically acceptable and chosen over MP in more than 75% of cases. The median SI score was 0.69 [0.41–0.84] and 0.73 [0.51–0.82] for MP and mCP, respectively (p >0.05).

**Conclusions:**

mCycle plans were comparable to clinical manual plans, more complex but accurately deliverable and registering a similar SI. Automated plans outperformed manual plans in blinded clinical choice.

## 1 Introduction

Recent studies focused on the implementation and commissioning of automatic tools in the typical radiotherapy workflow steps (1–3). The main characteristic of these tools is their mimicking of human planners’ interactions with the treatment planning system (TPS). Three paradigms have been exploited in commercially available auto-planning solutions: knowledge-based planning (KBP), protocol-based algorithms, and multicriterial optimization (MCO). The KBP (e.g., RapidPlan in Eclipse TPS, Varian Medical Systems, Palo Alto, California) is based on a library of clinically accepted, high-quality plans. KBP suggests how good a plan could be by comparing the new patient’s anatomy with the plan library, as a planner would learn from experienced colleagues’ suggestions (4). Protocol-based algorithms (e.g., Autoplanning in Pinnacle^3^ TPS, Philips Medical Systems, Fitchburg, Wisconsin) automatically repeat a known sequence of inverse planning actions, simulating the planner’s presence at the TPS (5). On the other hand, the MCO sequentially tries to get an organ at risk (OAR) spared as well as possible without compromising the target coverage by substituting the planner in the typical trial-and-error procedure. In particular, the MCO looks for optimal solutions belonging to the so-called Pareto’s surface, meaning that a plan cannot be further improved on any objective without degrading the results on at least one of the others. This surface navigation is done by the TPS in the *a priori* MCO, proposing only one planning solution respecting the listed requests (e.g., Monaco TPS, Elekta AB, Stockholm, Sweden). In the a-posteriori approach, the user can navigate between the generated multiple plans to choose the plan that best meets the clinical requests (e.g., Eclipse TPS, Varian, and Raystation TPS, RaySearch Laboratories AB, Stockholm, Sweden).

Together with the MCO, lexicographic optimization (LO) can be listed as a hierarchical optimization approach. LO is based on the imitation of the plan discussion process between radiation oncologists and planners, characterized by given clinical dose constraints, distribution evaluation, and compromise between conflicting planning goals (6). To do so, the given planning criteria are subdivided into constraints, which cannot be violated, and prioritized objectives with an assigned relevance order. Compared to MCO, in the LO sequential iterations, the obtained objective results are turned into constraints so that the following iterations cannot invalidate what has already been reached. The set of constraints, objectives, and priorities is called a Wish List (WL). The LO was first introduced and implemented at the Erasmus MC Cancer Institute of Rotterdam in the iCycle software (7). It is now implemented in the research version of Monaco TPS v5.59 combined with the *a priori* MCO as a research-available tool for photon beams and called mCycle (Elekta AB, Stockholm, Sweden). Although iCycle and mCycle are conceptually similar, their implementation is strongly different. The Rotterdam workflow is composed of two steps in the iCycle optimizer and in Monaco TPS, respectively. It starts with a fluence map optimization (FMO) in the iCycle multicriterial optimizer. The obtained distribution is the input to define a patient-specific Monaco template, which is subsequently used for final plan generation with the Monaco TPS. On the other hand, mCycle is completely embedded in the Monaco environment and no passages are needed. mCycle automated planning starts with a wish-list driven multicriterial FMO but the FMO dose distribution is directly input for a multi-criterial optimization of MLC segments answering again to the WL. The WL is defined by translating the physician’s main and secondary planning requests into a sequence of clinical (CC) and planning constraints (PC) and the following objectives. Usually, CC are the inviolable clinical requests (e.g., spinal cord maximum dose), and they are assigned a higher weight than PC. Objectives get weighted proportionally to their priority in the WL. Each constraint and objective must be translated into one or more cost functions associated with the contoured structures.

A completely new code has been written to embed this approach in the Monaco environment with a different mathematical solver, a different patient model, the typical Monaco cost functions, and a Monte Carlo Algorithm (XVMC) (8). The published experiences are mainly focused on iCycle application in several anatomic sites, while applications of the novel mCycle are reported only for head and neck, prostate and rectal cancer volumetric-modulated arc therapy (VMAT) treatment planning (8, 9), and prostate treatment on an MR-Linac (10). This is the first feasibility study of mCycle implementation for cervical cancer treatment. This pelvic anatomic site was chosen to study the possibility of reducing planner workload. Cervical cancer treatments are characterized by large and irregular-shaped targets that pose challenges to the generation of high-quality plans (11). They cover more than 10% of VMAT plans at our institution, contributing to the annual workload almost as much as prostate cancer treatments. This feasibility study thoroughly investigates mCycle performances to produce a plan quality at least comparable to accepted clinical manual plans obtained with clinical Monaco *a priori* MCO. The analysis included a comparison of plan dose distributions, complexity, delivery accuracy, and clinical acceptability. A plan quality score was introduced to globally assess manual and automatic plans, as suggested by previous studies claiming how powerful these indexes are to discern plan quality (12).

## 2 Materials and methods

### 2.1 Patient population

This retrospective planning study included a mono-institutional consecutive cohort of 24 cervical cancer patients previously treated with the VMAT technique between November 2019 and April 2022. All patients were treated at an Elekta VersaHD linear accelerator equipped with the Agility Multileaf Collimator (MLC, 160 leaves, 5 mm thickness, up to 6.5 cm/s, MU calibration 1 MU = 1 cGy). The main inclusion criterion was a prescription dose of 50 Gy in 25 fractions. To cover all the possible scenarios, the selected population included 9 patients who had undergone surgery and 15 patients who had not. This challenged the mCycle algorithm to manage different target volumes. The presence of a mono- or bilateral femoral prosthesis was considered an exclusion criterion. All patients underwent a CT simulation with a 3 mm slice thickness in the supine position, with rectum- and bladder-specific preparation instructions before the simulation and each treatment fraction. The originally segmented structure sets included clinical target volumes, CTVs (cervix, uterus if present, proximal vagina, and pelvic nodes), and OARs (rectum, bladder, small bowel, and femoral heads). These structure sets were used in the automatic re-optimization and the following data analysis. According to the institutional protocol, the planning target volume (PTV) was defined as the isotropic 7-mm expansion of the CTV. All patient-related information was deeply anonymized before conducting the research. The institutional review board denied the need for written informed consent from the participants as there was no impact on treatment and the applied patient data in this retrospective dosimetric planning study.

### 2.2 Manual treatment planning

All manual plans were generated with the clinical *a priori* MCO of the Monaco TPS (version 5.51.10). A 6-MV coplanar dual 330°-arc was optimized with up to 150 control points (CP), a minimum segment width of 1.0 cm, and highly smoothed fluence. A 3-mm dose grid and a 1%-statistical uncertainty per Monte Carlo calculation have been used. The manual optimization used the so-called MCO-constrained modality: the the PTV-related requests will be satisfied after the fulfillment of OAR cost functions because PTV and OAR cost functions were handled as first-order objectives and first-order constraints, respectively. The main limitation of this approach is the strong dependence on how the cost functions are manually defined, i.e., the defined parameters are manually modified, iteration by iteration, to modulate the PTV-OAR compromise in search of the best clinical plan. In MP, both Fluence Matrix Optimization (FMO, phase 1) and Segment Shape and Segment Weight Optimization (SSO and SWO, phase 2) phases have been performed with MCO. A final re-normalization of the dose distribution to reach the minimum PTV coverage or to fulfill the small bowel constraint was allowed. The minimum target coverage of V_95%_ >95% was requested with a D_1%_ <107%. Institutional OAR tolerance doses were rectum D_50%_ <44.7 Gy, bladder D_50%_ <57.3 Gy, small bowel V_45Gy_ <195 cm^3^, femoral heads D_5%_ <44.7 Gy (13–16). If it was not possible to respect the protocol constraints, minor or major deviations were discussed and accepted by the approving clinician.

### 2.3 mCycle auto-planning

As previously described, in mCycle, constraints and prioritized objectives are managed by the planner through the mCycle WL, which represents a dialogue between the radiation oncologist and the planner. The WL has to be tuned accordingly to the institution’s clinical protocol to obtain an optimal clinical plan in a single optimization process. As described by Hussein et al. (3), the tuning process is a multi-step iterative method on a subset of patients in which the current WL is evaluated in terms of the optimized plans. From a practical standpoint, the creation of a WL starts from a robust template of the manual planning process, following these simple guidelines:

- Identifying what the prescriptions are, whose violation would prevent the acceptance of the plans and indicate them as WL CC;
- Identifying those prescriptions that are normally inserted to determine dose gradients and indicating them as WL PC;
- If the priority is a minimal target coverage (for example, 95% of the prescription dose to 95% of the volume), assign this prescription as the first priority-objective
- Assign all subsequent priorities to the OARs according to the clinical relevance discussed with the radiation oncologist;
- If there is still room for optimization, assign lower-order priorities to ask again for certain OAR sparing or target coverage.

The user then iteratively acts on the type of cost function, their priority order, and their related goals. This iterative process continues on until the results satisfy the defined clinical protocol for a subset of patients without incurring the cost of not accurately delivering plans.

The WL is used in a two-pass automated lexicographic MCO during mCycle fluence optimization. During the first pass (FP), the fluence is optimized to sequentially get, for each defined cost function, a value lower than the requested goal. The obtained value is then used to constrain its cost function in the following second pass (SP). In the SP, all the objectives that were below their goal in the FP are further optimized till they reach their lowest possible value or till a specified “sufficient value” is reached. On the other hand, the objectives that were higher than their goal after the FP are constrained to the value reached during the FP. This sequential definition of new constrained values allows for the avoidance of repetitive manual interventions on the cost function parameters and for achieving the clinically desired plan in only one optimization.

The obtained optimized fluence map distribution is input for the following multi-criterial optimization of MLC segments using the new Pseudo-Gradient Descent Segment Shape Optimizer (PGDSSO), which, again, deals with the WL. Starting from the collection of beamlets and for each segment, it computes the benefit of including or excluding the i-th beamlet with an optimization method similar to gradient descent.

The WL definition requires initial tweaking using a subset of CTs and structure sets to get robust WL-producing automatic plans at least comparable to the retrospectively selected manual plans. To avoid any bias, the patients used to tune the WL have been excluded from the following analysis. The tuned WL has been exploited to automatically re-plan the final selection of treatment plans. The same arc configuration has been used with a 6-MV coplanar dual 330°-arc with up to 150 CP, 1-cm minimum segment width, highly smoothed fluence, 3-mm dose grid, and 3%-statistical uncertainty per CP in the Monte Carlo calculation. No further WL changes were allowed in this test phase. The only accepted manual intervention on the mCycle plans (mCP) was limited to minimal fine-tuning to obtain the minimum clinical acceptability, namely a second optimization reducing the minimum segment width to 0.75 cm or, as in MP, a final re-normalization of the dose distribution to reach the minimum PTV coverage or to fulfill the small bowel constraint. As indicated before, this normalization step is often done in the clinical manual routine to obtain minor adjustments in dose distributions, being careful to not invalidate plan deliverability (i.e., losing segments with a low number of monitor units (MU)).

### 2.4 Plan comparison

#### 2.4.1 Dosimetric comparison

To guarantee an unbiased comparison, all plans were recalculated using a statistical uncertainty of 0.5%. The MP and mCP PTV coverages have been compared in terms of the PTV V_100%_, V_95%_, and D_1%_. The dose distributions have been compared in terms of the conformality index (CI_95%_ and CI_50%_), defined by the ratio between the total volume covered by the specified dose (95% and 50% of the prescription dose) and the volume of the PTV. The analyzed OAR metrics were the mean doses (D_mean_), the rectum and bladder D_50%_, the bowel V_45Gy_, and the femoral heads D_5%_.

#### 2.4.2 Plan complexity and delivery accuracy

The two planning modes have also been analyzed in terms of the total number of monitor units (MU) and segments. Furthermore, the plan complexity has been quantified through the modulation complexity score (MCS), as defined by McNiven (17).

The plan delivery has been evaluated in terms of the agreement between the calculated and measured dose distributions, tested by performing a gamma analysis (ɣ). All plans have been recalculated on the CT scan of the Delta^4+^ phantom (ScandiDos, Uppsala, Sweden) using a 2-mm grid and a 0.5% statistical uncertainty. All plans, including MP, were delivered on the phantom on the same day to avoid daily delivery variations. The local ɣ has been performed with Scandidos software (version 1.00.0180). As established in the institutional QA protocol for conventional treatment plans, the gamma passing rate has been evaluated with a 3%/3 mm criteria (PR_33) neglecting any pixel registering a dose lower than 8% of the maximum dose (threshold). For sake of completeness, the 2%/2 mm criteria (PR_22), maximum gamma value (ɣ_max_), mean gamma value (ɣ_mean_), and confidence interval (ɣ_CI_ = ɣ_mean_ + 1.5 standard deviation) have been evaluated (18).

#### 2.4.3 Physicians’ blind choice

To clinically evaluate the mCycle results, two senior radiation oncologists (ROs) performed an independent, blind choice between MP and mCP, based on dose distribution, dose-volume histograms, and clinical objectives. All patients were anonymized and no information about the planning technique was given. Cohen’s kappa coefficient (k) has been used to measure inter-rater reliability.

#### 2.4.4 Plan quality score

**Figure 1:**
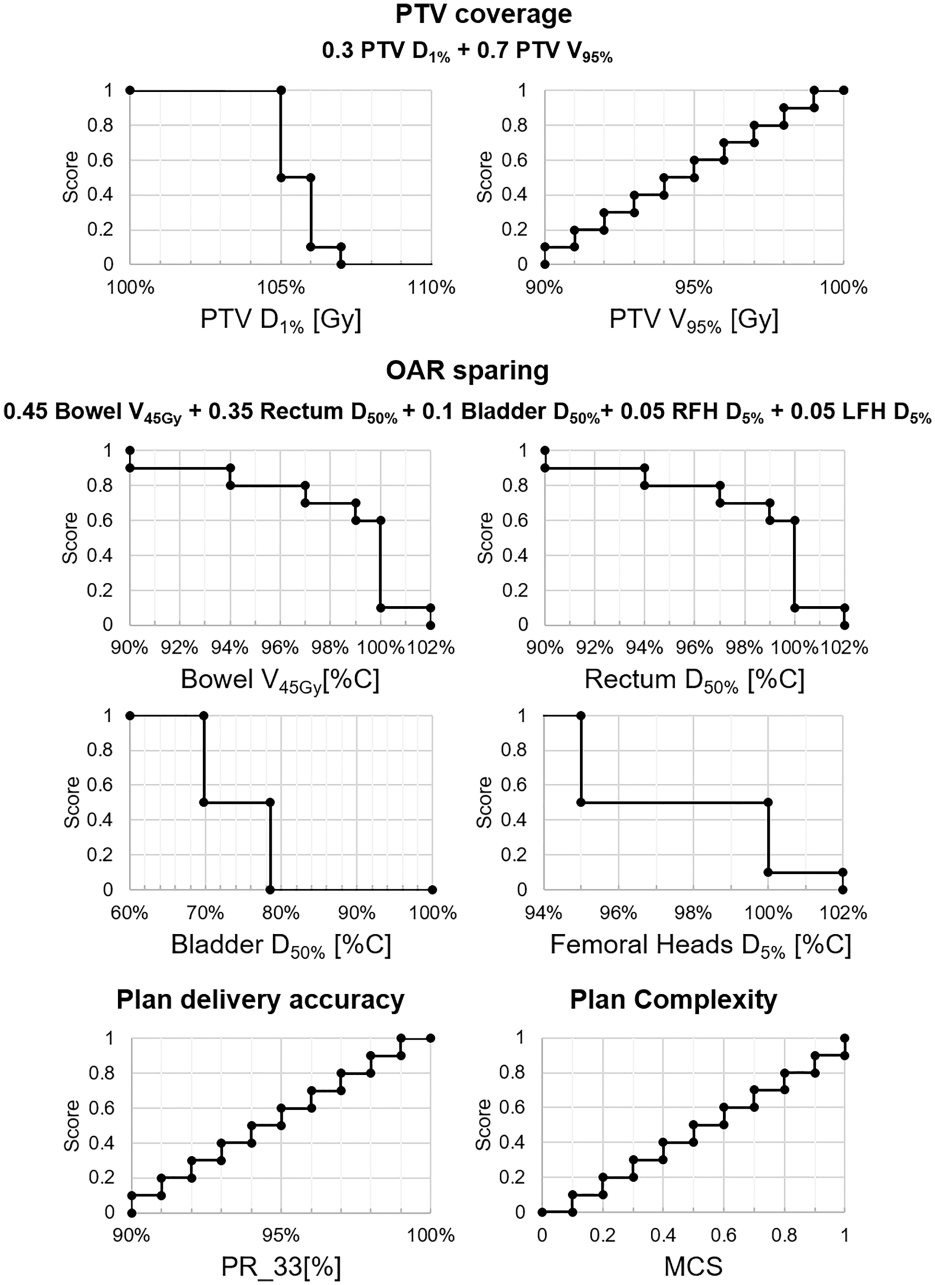
Scoring index components and definition. The weighting of target coverage and OAR sparing index components are reported in the formulae. Abbreviations: PTV, planning target volume; OAR, organ-at-risk; D_#_, dose received by the # % of contoured volume; V_#_, volume receiving more than # Gy; RFH, right femoral head; LFH, left femoral head; PR_33, 3%/3 mm gamma passing ratio; MCS, modulation complexity score.

The basic concept of a plan quality score was first introduced by Nelms (19), and it was here adapted to gather into a single number the plan evaluation process of our clinical team. The scoring index (SI) was defined as the quadratic mean of four sub-metrics representing target coverage, OAR-sparing, plan delivery accuracy, and plan complexity, each one of them ranging from 0 to 1 (**Figure 1**). The target coverage and OAR sparing sub-metrics are composed of the evaluated dose metrics as indicated in **Figure 1**. For each sub-metric, the score is proportional to the constraint fulfillment. For example, the PTV D_1%_ has a null score if it is higher than 107% as requested by the clinical protocol. The score increases as the PTV D_1%_ decreases.

### 2.5 Statistical analysis

Firstly, the normality test of Shapiro–Wilk has been performed on each sample of comparison metrics to establish whether to conduct the parametric t-test or the nonparametric Wilcoxon rank-sum test. The Bonferroni correction for multiple tests has been applied and the selected significance level has been set at 5% (p = 0.05). The variances of the two groups have also been compared with Bartlett’s test and Levene’s test (5) for normal and non-normal distributions, respectively. All statistical tests have been performed using Rstudio (2021.09.0).

## 3 Results

### 3.1 WL tweaking and automatic planning

The WL definition requested a tweak on four patients in five working days, acting on cost functions, their priority order, and their related goals. If a double PTV was present (PTV uterus and PTV pelvis), the PTV requests were simply doubled and kept both as first-priority objectives.

The final WL is presented in **Table 1**. The fulfillment of the bowel bag (CC), which violation implies plan rejection most of the time, is followed by dose gradient requests (PC). Finally, the objectives sequence asks for PTV coverage (first priority) and the iterative research of OAR doses as low as possible (following lower priorities).

**Table 1:** mCycle wish-list for auto-planning of cervical cancer (50 Gy in 25 fractions).

***Clinical Constraints***				
	***Structure***	***Cost Function*** ***(parameter)***	***Shrink margin (cm)***	***Limit***
	PTV	Quadratic Overdose (52 Gy)	/	<0.02 Gy
	Bowel bag	Overdose DVH (45 Gy)	/	<195.0 cm^3^
	External	Maximum Dose	/	53.4 Gy
***Planning Constraints***				
	***Structure***	***Cost Function*** ***(parameter values)***	***Shrink margin (cm)***	***Limit***
	External	Quadratic Overdose (50 Gy)	0.0	<0.1 Gy
	External	Quadratic Overdose (45 Gy)	0.3	<0.2 Gy
	External	Quadratic Overdose (40 Gy)	0.6	<0.2 Gy
	External	Quadratic Overdose (35 Gy)	0.9	<0.2 Gy
	External	Quadratic Overdose (25 Gy)	2.5	<0.3 Gy
***Objectives***				
***Priority***	***Structure***	***Cost Function*** ***(parameter values)***	***Shrink margin (cm)***	***Goal value (sufficient)***
1	PTV	Target EUD (0.5)		50.0 Gy
1	PTV	Target Penalty (99%)		50.0 Gy
2	Rectum	Parallel (40 Gy, *k* = 3)	0.3	<30.0%
2	Bladder	Parallel (40 Gy, *k* = 3)	0.3	<33.5%
3	Rectum	Serial (*k* = 15)		<46.0 Gy
3	Bowel bag	Parallel (40 Gy, *k* = 3)	0.3	<20.0%
3	Bowel bag	Serial (*k* = 15)		<43.0 Gy
3	Bladder	Serial (*k* = 15)		<47.0 Gy
4	Bowel bag	Overdose DVH (45 Gy)		<14.0% (7.0%)
4	Right femoral head	Serial (*k* = 15)		<38.0 Gy
4	Left femoral head	Serial (*k* = 15)		<38.0 Gy
5	External	Conformality		<0.75
6	Right femoral head	Serial (*k* = 1)		<30.0 Gy
6	Left femoral head	Serial (*k* = 1)		<30.0 Gy
7	Rectum	Serial (*k* = 1)		<30.0 Gy
8	Bladder	Serial (*k* = 1)		<35.0 Gy

Priority: order list according to which the objectives (cost functions) are optimized. Shrink margin: creates a buffer zone between the PTV and overlapping structures to avoid conflict between the applied cost functions of each of the structures. PTV, planning target volume; DVH, dose volume histogram; EUD, equivalent uniform dose.

The following automatic re-planning for the remaining 20 test patients took three working days. Excluding the contouring and the plan finalizing process, the median optimization and Monte Carlo calculation time can be estimated at 4 h and just over 1 h per MP and mCP, respectively. The manual fine-tuning was limited to six out of 20 plans (30%). A small re-normalization has been applied to get the minimum acceptable coverage or to satisfy the bowel constraint. In two cases, the re-normalization did not allow us to fulfill the clinical protocol bowel request and a re-optimization with a 0.75-cm minimum segment width was needed.

### 3.2 Plan comparison

#### 3.2.1 Dosimetric comparison

**Figure 2:**
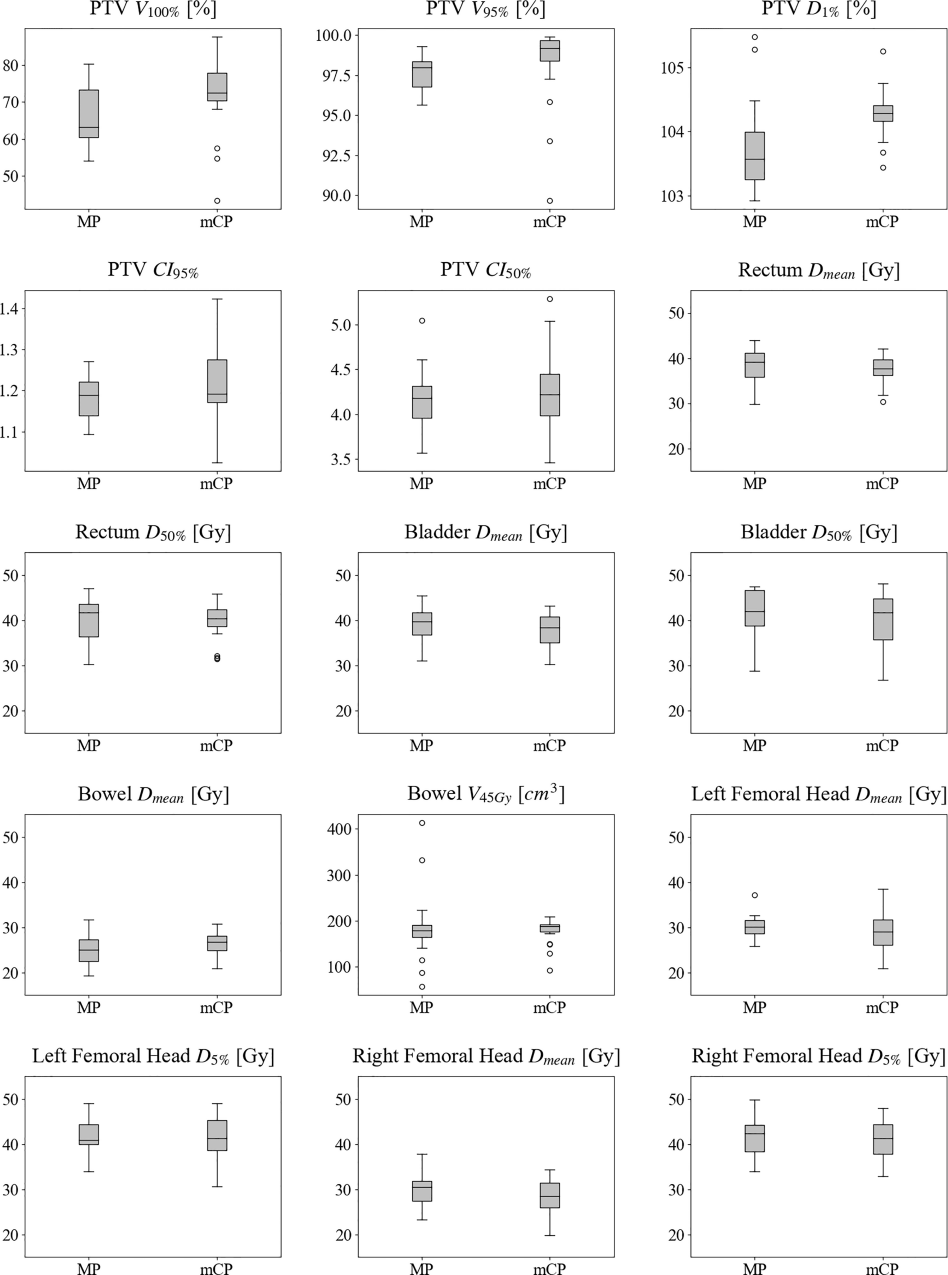
Box-and-whisker plots of computed dosimetric metrics for manual plans and mCycle plans. The box is delimited by the first (25%) and the third (75%) quartiles, and the bold line represents the median value. The whiskers point to the minimum and maximum data without counting boxplot outliers that in case there exist are represented with circles. An outlier is defined as a value exceeding the 1.5 interquartile range [1.5 (third–first quartile)]. MP, manual plans; mCP, mCycle plans; PTV, planning target volume; V_#_, volume receiving more than # Gy; D_#_, dose received by the # % of contoured volume; CI_#_, conformality index of the # dose; D_mean_, mean dose.

The selected patients registered a median PTV of 1,073.7 cm^3^ [608.4–1,453.9]. The MP and mCP dose results and their box-and-whisker plots are reported in **Table 2** and **Figure 2**. Statistically significant differences resulted in median values of target metrics, even if the significance remained only in D_1%_ once the multiple-tests correction is applied. Furthermore, the variance test showed a statistically significant difference for the PTV CI_95%_ with an associated mCP distribution larger than the MP one.

**Table 2:** Comparison of original manual plans and mCycle plans in terms of PTV and OAR dose metrics.

DOSE METRICS	MP	mCP	Wilcoxon or t test	Levene’s or Bartlett’s test
**PTV**				
V_100%_ (%) ^(2)^	63.3 [54.2–80.3]	72.4 [43.3–87.7]	**0.040**/1.000	0.597
V_95%_ (%) ^(2)^	98.0 [95.6–99.3]	99.2 [89.7–99.9]	**0.004/**0.108	0.391
D_1%_ (%) ^(2)^	103.6 [103.0–105.5]	104.3 [103.4–105.2]	**0.001**/**0.027**	0.066
CI_95%_^(1)^	1.2 [1.1–1.3]	1.2 [1.0–1.4]	0.218/1.000	**0.018**
CI_50%_^(1)^	4.2 [3.6–5.0]	4.2 [3.5–5.3]	0.379/1.000	0.376
**Bowel**				
V_45 Gy_ (cm^3^) ^(2)^	179.2 [56.5–414.0]	188.3 [92.6–209.0]	0.344/1.000	0.080
D_mean_ (Gy) ^(1)^	25.0 [19.3–31.7]	26.7 [20.8–30.8]	0.142/1.000	0.518
**Rectum**				
D_50%_ (Gy) ^(1)^	41.7 [30.2–47.0]	40.3 [31.4–45.8]	0.713/1.000	0.430
D_mean_ (Gy) ^(1)^	39.1 [29.7–44.0]	37.7 [30.4–42.1]	0.404/1.000	0.273
**Bladder**				
D_50%_ (Gy) ^(2)^	42.0 [28.8–47.4]	41.6 [26.7–48.1]	0.583/1.000	0.631
D_mean_ (Gy) ^(1)^	39.7 [30.9–45.4]	38.4 [30.3–43.1]	0.293/1.000	0.661
**Left femoral head**				
D_5%_ (Gy) ^(1)^	40.9 [33.9–49.0]	41.3 [30.5–48.9]	0.872/1.000	0.239
D_mean_ (Gy) ^(1)^	30.1 [25.8–37.2]	29.0 [20.9–38.4]	0.186/1.000	0.060
**Right femoral head**				
D_5%_ (Gy) ^(1)^	42.3 [34.0–49.8]	41.3 [32.8–47.9]	0.734/1.000	0.973
D_mean_ (Gy) ^(1)^	30.5 [23.2–37.8]	28.4 [19.8–34.3]	0.126/1.000	0.416

MP, manual plans; mCP, mCycle plans; PTV, planning target volume; V_#_, volume receiving more than # Gy; D_#_, dose received by the # % of contoured volume; CI_#%_, conformality index of the #% of the prescription dose; D_mean_, mean dose; ^(1)^, Gaussian distribution; ^(2)^, not normal distribution. In Wilcoxon and t test column, non-corrected and Bonferroni-corrected p-values are reported (p-value/corrected p value). Bold: Statistical significance (p <0.05). Median values and ranges are reported.

On the other hand, results in OAR sparing showed comparable performances in all considered metrics. A slight decrease in OAR metrics can be observed in mCP, but it did not register statistical significance. It is worth noticing the mCP high repeatability of the bowel V_45Gy_ with an extremely narrow boxplot just below the constraint.

**Figure 3:**
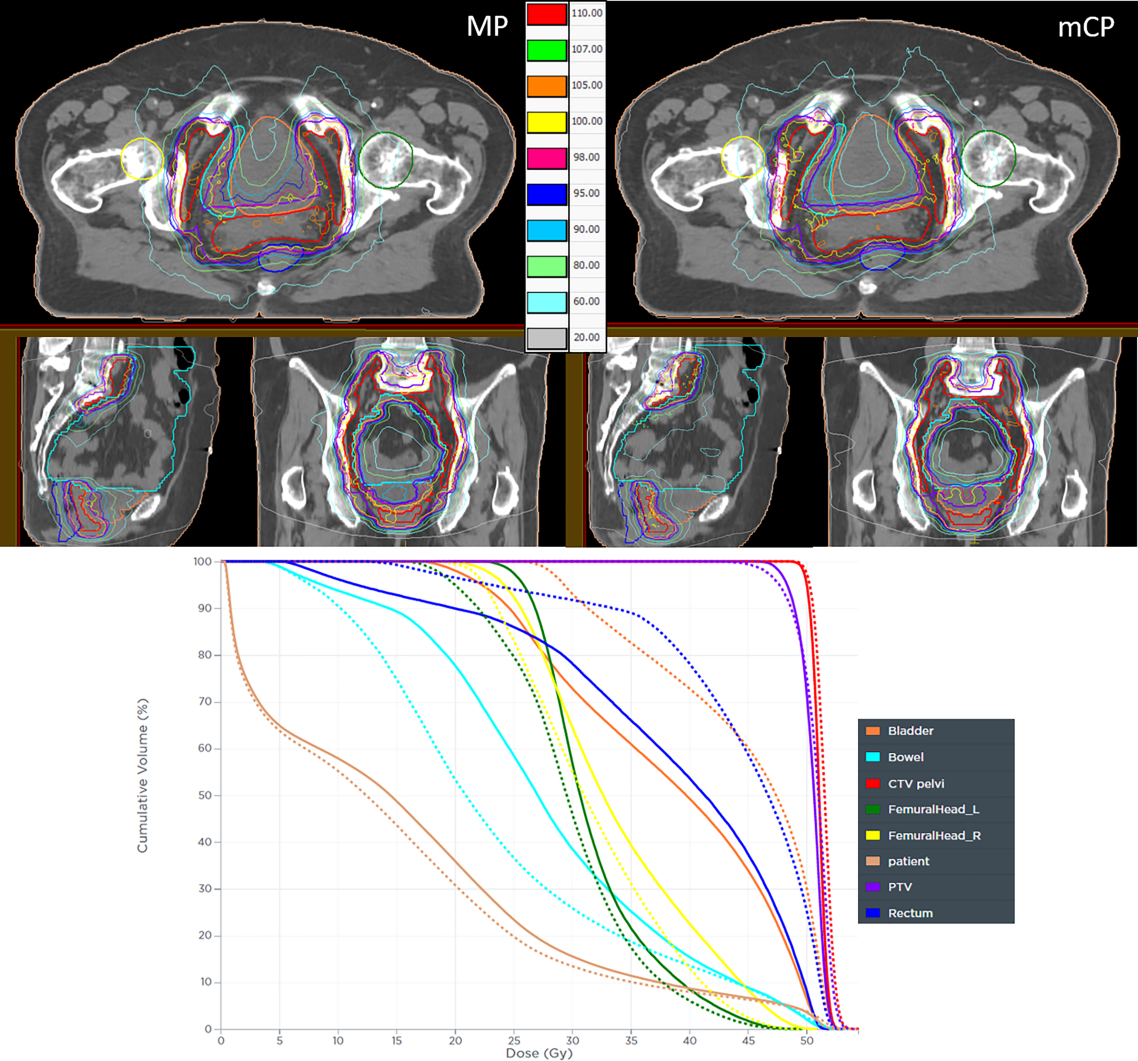
Dose distribution comparison of a manual plan (MP) and a mCycle plan (mCP). The isodose color legend is reported while the contoured structures are CTV (red), PTV (purple), rectum (blue), bladder (orange), bowel (cyan), right femoral head (yellow), left femoral head (green), and patient (pink). The DVH curves are reported as solid lines for mCP and dotted lines for MP.

The dose distributions for a representative patient are graphically reported in **Figure 3**. This shows a slight increase in the PTV coverage and a significant improvement in rectum and bladder sparing. The bowel V_45 Gy_ respected the clinical constraints in both plans, but it is worth noticing the greater extent of the low doses in mCP than in MP.

#### 3.2.2 Complexity and delivery accuracy comparison

**Figure 4:**
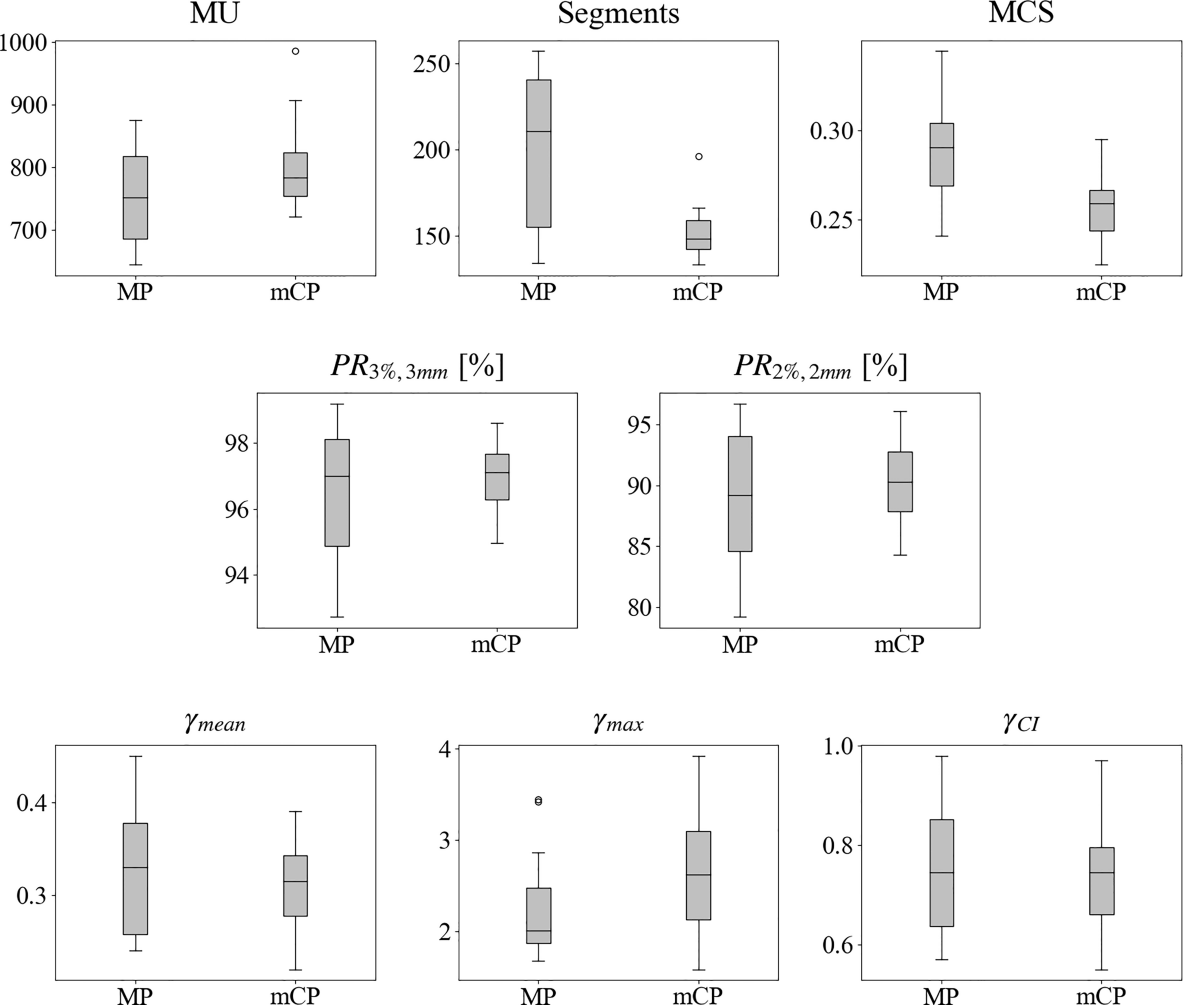
Box-and-whisker plots of computed complexity and delivery metrics for manual plans (MP) and mCycle plans (mCP). The box is delimited by the first (25%) and the third (75%) quartiles, and the bold line represents the median value. The whiskers point to the minimum and maximum data without counting boxplot outliers that in case there exist are represented with circles. An outlier is defined as a value exceeding the 1.5 interquartile range (1.5 (third–first quartile)). Abbreviations: MP, manual plans; mCP, mCycle plans; MU, monitor units; MCS, modulation complexity score; PR_33, 3%/3 mm gamma passing ratio; PR_22, 2%/2 mm gamma passing ratio; ɣ_mean_, mean gamma index value; ɣ_max_, maximum gamma index value; ɣ_CI_, gamma confidence interval.

Plan complexity and delivery results and their box-and-whiskers plots are reported in **Table 3** and **Figure 4**, respectively. The mCP registered a MU slight increase and a statistically significant MCS decrease (p <0.001). On the other hand, a decrease in the number of segments has been registered thanks to the novel PGDSSO. A significant narrowing of data variance is registered for the MCS and number of segments. The increased complexity had no effect on plan delivery accuracy, as shown by the gamma passing ratios reported in **Table 3** with their p-values.

**Table 3:** Comparison of original manual plans and mCycle plans in terms of plan complexity and plan delivery. Median values and ranges are reported.

**PLAN COMPLEXITY**	**MP**	**mCP**	**Wilcoxon or t test**	**Levene’s or Bartlett’s test**
MCS^(1)^	0.29 [0.24–0.34]	0.26 [0.23–0.30]	**<0.001/0.002**	**0.047**
MU ^(2)^	751.2 [644.1–875.2]	783.5 [721.2–985.1]	0.086/1.000	0.188
Segments ^(2)^	211 [134–257]	148 [133–196]	**0.001/0.027**	**<0.001**
**PLAN DELIVERY ACCURACY**	**MP**	**mCP**	**Wilcoxon or t test**	**Levene’s or Bartlett’s test**
PR (3%/3 mm) (%) ^(1)^	97.0 [92.7–99.2]	97.1 [95.0–98.6]	0.441/1.000	**0.018**
PR (2%/2 mm) (%) ^(1)^	89.2 [79.2–96.7]	90.3 [84.3–96.1]	0.331/1.000	**0.031**
ɣ_mean_^(2)^	0.33 [0.24–0.45]	0.32 [0.22–0.39]	0.498/1.000	0.055
ɣ_max_^(2)^	2.01 [1.67–3.44]	2.62 [1.58–3.92]	0.079/1.000	0.190
ɣ_CI_^(1)^	0.75 [0.57–0.98]	0.75 [0.55–0.97]	0.911/1.000	0.274

MP, manual plans; mCP, mCycle plans; MCS, modulation complexity score; MU, monitor units; PR, gamma passing ratio; ɣ_mean_, mean gamma index value; ɣ_max_, maximum gamma index value; ɣ_CI_, gamma confidence interval; ^(1)^, Gaussian distribution; ^(2)^, not normal distribution. In Wilcoxon and t test column, non-corrected and Bonferroni-corrected p-values are reported (p-value/corrected p-value). Bold: Statistical significance (p <0.05).

#### 3.2.3 Blind choice results

Out of 20 patients, all manual and automated plans were considered clinically acceptable. Only two MPs presented a major deviation from the protocol criteria due to unfavorable anatomy.

Other minor deviations in OAR constraints were clinically accepted (2 MP and 3 mCP). The ROs chose the mCP over the MP in 75.0% and 80.0% of cases, with a moderate agreement (k = 0.51). The preferred MP plans registered a slightly better OAR spare with lower PTV coverage.

#### 3.2.4 Plan scoring

The target coverage, OAR sparing, plan delivery accuracy, and plan complexity median scores for MP and mCP were 0.90, 0.79, 0.75, 0.20, and 1.00, 0.81, 0.80, and 0.20, respectively. The median SI was 0.69 [0.41–0.84] and 0.73 [0.51–0.82] for MP and mCP, respectively. On a single patient-basis, the mCP median score variation with respect to MP was 5.8% [−17.0% to +72.0%]. No statistically significant difference was registered between the single metrics and the SI distributions. All data are listed in **Table 4**.

**Table 4:** Comparison of plan scoring index and its sub-metrics for plans and mCycle plans. Median values and ranges are reported.

SUBMETRIC*	MP	mCP	Wilcoxon	Levene’s test
Target coverage	0.90 [0.57–1.00]	1.00 [0.30–1.00]	0.013/0.195	0.722
OAR sparing	0.79 [0.32–1.00]	0.81 [0.05–1.00]	0.507/1.000	0.135
Plan delivery accuracy	0.75 [0.30–1.00]	0.80 [0.60–0.90]	0.466/1.000	**0.002**
Plan complexity	0.20 [0.20–0.30]	0.20 [0.20–0.20]	**0.004**/0.060	**0.003**
**SCORE INDEX**	0.69 [0.41–0.84]	0.73 [0.51–0.82]	0.159/1.000	0.302

*All metrics showed a not normal distribution. Abbreviations: MP, manual plans; mCP, mCycle plans. Non-corrected and Bonferroni-corrected p-values are reported (p-value/corrected p-value). Bold: Statistical significance (p <0.05).

## 4 Discussion

To the knowledge of the authors, this work is the first feasibility study of mCycle implementation in the VMAT planning of cervical cancer treatment. This novel tool differs from iCycle by exploiting Monaco cost functions, a new mathematical solver, and a new patient model, providing a single solution in which LO and MCO are coupled with Monte Carlo calculation. This qualitative and quantitative comparison with the clinically accepted plans generated by experienced medical physicists included a quantitative scoring of global plan quality. Results proved the mCycle capability to generate plans at least comparable to manual plans with a strongly limited manual plan fine-tuning. The number of patients needed to tune the WL is not defined in the literature, but the presented results are in line with Bijman et al.’s (8) experience on other anatomic sites. Furthermore, the automatic re-planning took only three working days to obtain 20 clinically acceptable and deliverable mCP, showing how mCycle would strongly reduce planners’ workload in the clinical routine of cervical treatment planning. In terms of dosimetric comparison, mCP was comparable to MP obtaining a target coverage increase. The registered increase of the PTV D_1%_ showed statistical significance, although not clinically relevant and respecting the institutional protocol. These results were obtained with a very narrow distribution of bowel-sparing results, proving the strength and repeatability of LO when a clinical constraint is given. It is worth noticing that the blind choice revealed that slightly lower OAR doses or a smaller low dose extent in MP, as shown in **Figure 3**, was preferred to the extremely high target coverage of the opposing mCP. The analysis of plan complexity and delivery accuracy proved that mCycle generates more complex plans even if the implementation of a new segment shape optimization led to a lower number of segments. The newer PGDSSO is faster and more efficient in merging similar segments than the previous algorithm (used in the MP), thus resulting in a lower global number of segments while keeping the modulation degree as high as needed by the plan. Nevertheless, the gamma analysis results showed that the accuracy of plan delivery was preserved and guaranteed. These results are limited to the available sample size, which was strongly dependent on the inclusion criteria. Further investigations will be needed to confirm these results on a larger dataset (20).

The mCycle capability to mimic manual planning has thus been verified in an anatomic site only investigated in a few other auto-planning experiences reported in the literature. Hussein et al. (21) and Tinoco et al. (22) have reported the KBP capability to produce IMRT and VMAT treatment plans with comparable OAR sparing and better conformity than the original clinically accepted plans. Sharfo et al. investigated IMRT versus VMAT strategies for cervical cancer with their in-house Erasmus-iCycle optimizer (23) and demonstrated that the plan quality of automatically generated plans was superior to manually-generated plans (24).

Comparing this mCycle implementation with the published results on KBP (11, 21, 22, 25, 26) the main difference can be identified in the KBP need for a model optimization based on a high-quality plan library (21). As claimed by Cilla (5), KBP mainly depends on the model strength, i.e., on the original plan quality and the correct identification of plan outliers. On the other hand, mCycle asks for a WL optimization, which appears very simple and intuitive, resembling a template optimization that can be easily learned by any Monaco user. Furthermore, if a new clinical protocol is introduced, KBP needs a new database of high-quality manually generated plans. On the contrary, mCycle and the Autoplanning of Pinnacle allow implementing automatic plan generation only adapting the WL or the Pinnacle technique, respectively, using the very first patients’ CT scans and structures.

In this context of protocol updates, a single dose prescription WL can be considered to be a limitation. In fact, lymph node boosts are often included in external beam radiotherapy. Despite the capabilities of mCycle to deal with multiple dose prescriptions, in the current study, we only dealt with a single dose prescription. As it has already been demonstrated by other authors in head and neck cases (9) or prostate with simultaneous boost (10), multiple prescriptions are easily handled by mCycle WL exploiting the LO capability to spare OAR as much as possible without affecting a higher dose target coverage: the PTV coverage request should be doubled and differentiated for the PTV boost, and the goal values of OAR cost functions coherently adapted. This would lead to fine-tune the presented WL on a different subset.

Furthermore, three considerations on the WL optimization can be done. As already mentioned, even if mCP were all defined as clinically acceptable, their extremely wide target coverage was sometimes ranked lower than a slightly better OAR sparing in MP. Secondly, the planner’s manual intervention has been reduced to a very small number of clicks, but 10% of mCP asked for a second optimization. Finally, the compared manual plans were obtained in the clinical routine by expert planners with limited available time, while Sharfo et al. proved an LO plan quality superior to manual plans even when generated by an expert planner without time pressure (24).

These considerations led to a further investigation into the possible WL tuning to explore the possibility of further sparing the surrounding OARs without any manual intervention and challenging a planning expert with no planning time limits. To do so, the LO gives the possibility to introduce multiple requests and priority levels, while the presented WL is now very straightforward. Further studies are ongoing to investigate the possibility to tune a second more complex WL highly demanding on OAR sparing, increasing the plan complexity, and, of course, the risk to affect plan deliverability. Having two WLs, it would be possible to choose the preferred compromise between dose distribution and plan complexity for each patient.

The RATING guidelines for treatment planning studies were used by three authors (PC, MCD, and BB) to independently score this study (27). The RATING scores were 93%, 89%, and 94%.

## 5 Conclusions

This is the first retrospective feasibility study on mCycle planning for cervical cancer treatment. The presented results showed that mCycle is an effective tool to generate automatic, high-quality VMAT treatment plans according to the cervical treatment institutional protocol. In fact, this comprehensive dosimetric and clinical evaluation showed that mCycle plans were comparable to clinical manual plans at the dosimetric comparison, more complex but equally deliverable. In addition, they registered a slightly higher global quality score. Furthermore, the needed planning time has been reduced by nearly a quarter. Finally, automated plans outperformed manual plans in blinded clinical scoring. As soon as it becomes commercially available, its implementation into the clinical routine will lead to reduced planning workload and dosimetric and clinical advantages. Future studies will broaden its use to other anatomic sites.

## Data availability statement

The raw data supporting the conclusions of this article will be made available by the authors, without undue reservation.

## Author contributions

ST was the lead author, who participated in study design, data collection, data and statistical analysis, manuscript drafting, table/figure creation, and manuscript revision. PC contributed to study design, participated in data collection and analysis, manuscript drafting, table/figure creation, study rating, and manuscript revision. RP participated in data collection, data analysis, and manuscript revision. GM participated in data collection and analysis, and manuscript revision. MD participated in table/figure creation, study rating, and manuscript revision. BB participated in study rating and manuscript revision. VF and DP aided in data analysis and manuscript revision. SM and EB participated in the plan blind choice and manuscript revision. SA is a senior author who aided in data analysis and manuscript revision. EP is a senior author who aided in the study design, contributed to statistical analysis, and revised the manuscript. All authors contributed to the article and approved the submitted version.

## Conflict of interest

RP serves as Director of Clinical Science at Elekta AB.

The remaining authors declare that the research was conducted in the absence of any commercial or financial relationships that could be construed as a potential conflict of interest.

## Publisher’s note

All claims expressed in this article are solely those of the authors and do not necessarily represent those of their affiliated organizations, or those of the publisher, the editors and the reviewers. Any product that may be evaluated in this article, or claim that may be made by its manufacturer, is not guaranteed or endorsed by the publisher.

## Abbreviations

LO, lexicographic optimization; MCO, multicriterial optimization; WL, wish list; MP, manual plans; mCP, mCycle plans; SI, scoring index; OAR, organ-at-risk; PTV, planning target volume; TPS, treatment planning system; KBP, knowledge-based planning; VMAT, volumetric-modulated arc therapy; CTV, clinical target volume; CP, control points; FMO, Fluence matrix optimization; SSO, segment shape optimization; SWO, segment weight optimization; CC, clinical constraint; PC, planning constraint; FP, first pass; SP, second pass; PGDSSO, pseudo-gradient descent segment shape optimizer; MUs, monitor units; MCS, modulation complexity score; ROs, radiation oncologists.
